# FASB: an integrated processing pipeline for Functional Analysis of simultaneous Spinal cord-Brain fMRI

**DOI:** 10.21203/rs.3.rs-3889284/v1

**Published:** 2024-01-30

**Authors:** Shahabeddin Vahdat, Caroline Landelle, Ovidiu Lungu, Benjamin De Leener, Julien Doyon, Fatemeh Baniasad

## Abstract

Simultaneous functional magnetic resonance imaging (fMRI) of the spinal cord and brain represents a powerful method for examining both ascending sensory and descending motor pathways in humans *in vivo* . However, its image acquisition protocols, and processing pipeline are less well established. This limitation is mainly due to technical difficulties related to spinal cord fMRI, and problems with the logistics stemming from a large field of view covering both brain and cervical cord. Here, we propose an acquisition protocol optimized for both anatomical and functional images, as well as an optimized integrated image processing pipeline, which consists of a novel approach for automatic modeling and mitigating the negative impact of spinal voxels with low temporal signal to noise ratio (tSNR). We validate our integrated pipeline, named FASB, using simultaneous fMRI data acquired during the performance of a motor task, as well as during resting-state conditions. We demonstrate that FASB outperforms the current spinal fMRI processing methods in three domains, including motion correction, registration to the spinal cord template, and improved detection power of the group-level analysis by removing the effects of participant-specific low tSNR voxels, typically observed at the disk level. Using FASB, we identify significant task-based activations in the expected sensorimotor network associated with a unilateral handgrip force production task across the entire central nervous system, including the contralateral sensorimotor cortex, thalamus, striatum, cerebellum, brainstem, as well as ipsilateral ventral horn at C5-C8 cervical levels. Additionally, our results show significant task-based functional connectivity between the key sensory and motor brain areas and the dorsal and ventral horns of the cervical cord. Overall, our proposed acquisition protocol and processing pipeline provide a robust method for characterizing the activation and functional connectivity of distinct cortical, subcortical, brainstem and spinal cord regions in humans.

## Introduction

A plethora of evidence suggests that many of the movement disorders, such as amyotrophic lateral sclerosis, multiple sclerosis, Parkinson's disease, stroke, spinal cord injury, dystonia, and cerebral palsy impair both the brain and spinal cord circuits (Pierrot-Deseilligny and Burke, 2012; Raudino and Leva, 2012; Wahl and Schwab, 2014). However, human neuroimaging studies with patients affected by these disorders have mainly focused on characterizing the brain circuit dysfunction and deficits, while disregarding the critical role of the spinal cord circuitry and brain-spinal cord pathways. Simultaneous functional magnetic resonance imaging (fMRI) of the spinal cord and brain provides an unprecedented opportunity to address this limitation by allowing the assessment of the ascending sensory and descending motor pathways functioning in humans *in vivo* (Kinany et al., 2022; Landelle et al., 2021; Tinnermann et al., 2021). By employing this combined approach, researchers can investigate not only the neural activations at multiple levels of the central nervous system (CNS), including cortical, subcortical, brainstem, and spinal cord, but also functional interactions among these structures, thereby gaining valuable insights into the neural mechanisms underlying motor and sensory processing. Nevertheless, the imaging parameters and processing pipeline associated with this simultaneous neuroimaging approach are still in the early stages of development. In this study, we introduce an optimized acquisition protocol including the required anatomical and functional images, as well as an integrated analysis pipeline, named Functional Analysis of simultaneous Spinal cord-Brain fMRI (FASB). The proposed pipeline aims to improve the current challenges associated with the processing of spinal cord fMRI data under simultaneous spinal cord-brain acquisition in three domains. These improvements include motion correction, registration to the template, and removing the effects of large susceptibility artifacts, often exacerbated at the spinal disk levels.

Prior work using simultaneous spinal cord-brain fMRI has revealed that task- and context-dependent dynamical interactions between brain regions and the spinal cord subserve various sensory and motor functions. In the last decade, the optimization of acquisition protocols at high-field strength (Barry et al., 2018b), as well as the development of multi-slab pulse sequences designed to improve the acquisition of selective fields-of-view (FOVs), combined with advanced shimming procedures (Finsterbusch et al., 2013, 2012; Islam et al., 2019) have significantly enhanced the quality of spinal cord-brain fMRI. Using these advanced image acquisition and shimming techniques, we have recently demonstrated the existence of integrated spinal cord and brain resting-state networks that follow neuroanatomical principles governing the ascending and descending pathways in humans (Vahdat et al., 2020). Despite clear advancement in spinal cord fMRI thanks to these new techniques, there is still work needed to improve image quality due to all the challenges associated with functional imaging of the spinal cord (Landelle et al., 2021).

The application of spinal cord fMRI is starting to augment our brain-centered view of neurological disorders, by investigating the role of spinal cord circuit function in movement disorders, *e.g.* in Parkinson's disease (Landelle et al., 2023). Yet studying the function of ascending and descending pathways requires the application of simultaneous fMRI methods, which has been limited in clinical populations due to a number of technical challenges (Landelle et al., 2021). These include the complex acquisition procedures that can achieve both a reasonable signal to noise ratio (SNR), and a high spatial resolution for distinguishing spinal cord quadrants, further constrained by the scanning time considerations. The goal of this study is to propose a simple acquisition method that can be implemented in clinical populations, together with an integrated processing pipeline to standardize the procedures for simultaneous spinal cord-brain fMRI.

The spinal cord toolbox (SCT) (De Leener et al., 2017) has offered optimized set of tools for preprocessing of the spinal cord MRI data, including slice-wise motion correction, automatic segmentation of the spinal cord using convolutional neural networks (Gros et al., 2019), and nonlinear slice-wise registration to the template (De Leener et al., 2018). However, the combined acquisition of the spinal cord and brain provides new opportunities, as well as challenges, which can benefit from a customized processing pipeline. For example, the motion correction procedure implemented in the SCT is solely based on the spinal cord FOV, and can thus be improved by incorporating information from the acquired brain FOV. Also, large spatial distortions due to exacerbated susceptibility artifacts at the disk levels in BOLD imaging limit the efficacy of non-linear transformations for registration. Importantly, this may cause signal loss in the EPI data and may degrade SNR in some of the spinal cord slices. These low tSNR voxels practically degrade the group-level results by adding noisy voxels to the group-level averaging. Yet, there are two solutions to the latter problem. One approach is to remove the low SNR voxels from the participant's spinal cord mask. However, the voxels included at the group-level analysis should be present in the mask of every single individuals. Because low-SNR voxels appear at different locations across participants (depending on neck curvature, surrounding tissues, etc.), a large number of voxels will be removed from the group-level analysis, which makes this approach impractical. Another approach is to remove the effect of these low-SNR voxels statistically from the group-level analysis, by incorporating participant-specific voxel-wise confounds in the group-level regression model. In this study, we employ the latter idea and propose a new approach that features automated modeling and identification of spinal cord voxels with low temporal SNR in each participant, followed by removing their effects statistically at the group-level analysis without the need to exclude those voxels from the entire group.

To evaluate the efficacy of the proposed pipeline, FASB, we collected simultaneous spinal cord-brain fMRI data during the performance of a motor control task (unilateral handgrip force control), as well as during resting-state conditions. First, we compared the performance of the proposed algorithm for motion correction, registration to the spinal cord template, and physiological noise removal, to the current methods used in the spinal cord fMRI analysis. We then reported the activation maps in the brain, brainstem and spinal cord using FASB and other analysis methods during the motor control task. We hypothesized that the subject's performance of this unilateral handgrip task would significantly increase the level of activity in the ipsilateral ventral horn at the C6-C8 cervical levels (Pierrot-Deseilligny and Burke, 2012), as well as in the contralateral primary and secondary sensorimotor cortical areas, striatum, thalamus, ipsilateral cerebellar cortex (Doyon et al., 2018; Vahdat et al., 2011), and ipsilateral motor-related brainstem nuclei, including pontine and medullary reticular formation (Arber and Costa, 2022; Ruder and Arber, 2019). We further assessed the functional connectivity of the cervical dorsal and ventral horns with various sensory and motor areas of the brain and brainstem. Our results demonstrate that the proposed acquisition protocol and processing pipeline (FASB) outperforms other methods commonly used in spinal cord fMRI analysis, and provides a robust method for accurately characterizing the activation and functional connectivity of distinct cortical, subcortical, brainstem and spinal cord regions in humans in vivo.

### Methods

## Participants

This study was reviewed and approved by the ethics committee at the University of Florida, which adhered to the Declaration of Helsinki. All participants signed an informed consent form prior to participating in the study and were debriefed and compensated at the end of the experiment. Fifteen young, right-handed, healthy adults (10 females, mean age = 22.7 years, SD = 4.0) were selected to take part in this study based on the following exclusion criteria: presence of a history of neurological and psychiatric diseases, of any motor-system complication, the current use of any medication, and presence of any MRI-incompatible object in the body. An additional twenty-one healthy participants were recruited for resting-state acquisition (12 females, mean age = 31.4 years, SD = 7.1). These participants fulfilled the inclusion criteria previously described and the data acquisition was approved by the ethics committee at the University of Montréal, which also adheres to the Declaration of Helsinki. Due to poor fMRI image quality, resting-state data from two participants from this sample were not included in analyses, and data from another participant was also excluded due to missing physiological noise measurements. Thus, the resting-state sample included in the final analyses consisted of 18 healthy participants (mean age 32.39 (SD = 13.19), F/M = 11/7).

## Motor control task

Participants who performed a motor control task in the scanner were asked to produce isometric hand grip force with their right hand using an MR-compatible fiber-optic force transducer. Prior to the MRI scan, participants were first trained on the task, and the grip maximum voluntary contraction (MVC) was measured. Specifically, they were asked to produce unilateral grip force using their right hand by opposing their thumb, index, and middle fingers against the force transducer (Archer et al., 2018, 2016; Burciu et al., 2016). Two runs were completed in a block-design paradigm. Each run consisted of 9 blocks of 15 sec force control, followed by 25 sec rest periods (6 min and 25 sec in total). Throughout the run, 2 bars were displayed on a video monitor that participants saw through a mirror mounted on the head coil: a target bar and a force bar. A change in color of the force bar was used to cue participants to either push or release the force sensor. Green was a go signal for producing and sustaining force, while red indicated a rest period. The target bar was set at 10, 20, or 30% of MVC for each participant (3 blocks at each level, pseudo-randomly ordered), while the force bar moved in the vertical plane according to the force output. During the go signal, participants were required to adjust their grip force levels to match the force and target bars. Force was measured using custom-designed load cells (Vaillancourt et al., 2003) and a Micron Optics fiber optic interrogator.

## MRI data acquisition

**Motor control task dataset**. A 3T Siemens Prisma scanner equipped with a 64-channel head/neck coil was used for imaging subjects. We acquired functional magnetic resonance imaging (fMRI) data, covering the whole brain and cervical spinal cord (first cervical, C1, to first thoracic, T1, vertebrae), using blood oxygenation level dependent (BOLD) contrast via a simultaneous multi-slice (SMS) echo-planar imaging (EPI) sequence. Participants laid on the scanner table in a supine position with their head and neck fully supported using foam pads to minimize their motion. We used the neck and brachial plexus SatPad™ sets (SatPad, clinical imaging solutions) around the neck to minimize inhomogeneity artifacts and enhance spinal cord functional image quality. Based on previous work in the lab, we recommend their use in fMRI studies that target the spinal cord. Participants were placed in the scanner such that the mid-chin level was in the scanner's isocenter, corresponding to C2-C3 vertebral level on the axial plane. The following parameters were used for EPI acquisitions: 72 axial slices angled orthogonal to the cord at C5 vertebral level, in-plane resolution: 1.6x1.6 mm$^2$, matrix size: 120x120, 50% phase oversampling, slice thickness: 4 mm (no gap), TR: 1.85 s, 210 volumes, TE: 27 ms, multiband acceleration factor: 3, GRAPPA

factor: 2, 0.875 partial Fourier encoding, FA: 70˚, PE direction: A-P. Additionally, 2 saturation pulses were applied in a V-shaped configuration to minimize ghosting and inflow artifacts related to blood flow in the major cervical vessels.

A 3D-MPRAGE sequence was used to acquire T1-weighted anatomical images covering the entire head as well as neck down to the T3 vertebral level using the following parameters: field of view (FOV): 192×240×320 $mm^3$, sagittal slices; TR: 2300 ms, TE: 3.41 ms, and resolution: 1×1×1 $mm^3$. In addition, a multi-echo data image combination (MEDIC) sequence was used to acquire a T2*-weighted (T2w) anatomical image using the following parameters: axial slices = 36, slice thickness = 4 mm (no gap), in-plane resolution: 0.75×0.75 $mm^2$, matrix size: 256×256, TR: 1280 ms, TE: 18, 20, 21, and 22 ms; FA: 20˚, and GRAPPA acceleration factor: 2. Although the orientation and the size of slices were matched between the T2w and EPI images, the T2w image had fewer slices, covering only the cervical cord to keep the acquisition time down to 12 min. Importantly, the position of the T2w image FOV in the dorsocaudal orientation was set such that the position of the most caudal slice matches that of the EPI image.

**Resting-state dataset.** Data were also acquired using a 3-Tesla MRI Scanner (Magnetom-Prisma, Siemens, Erlangen, Germany) equipped with a 64-channel head (1–7 elements active) and neck coil (1–2 elements active). T2-weighted images were collected in the transverse orientation with the following parameters: TR = 33ms; TE = 14ms; FOV = 224 mm x 258 mm; flip angle = $5^o$; in-plane voxel resolution = 0.35 x 0.35 $mm^2$, slice thickness = 2 mm. A total of 112 slices were acquired, spanning from the top of the cerebellum to the upper thoracic region (approximately at T1), thereby encompassing the entirety of the cervical spinal cord. Resting-state functional images were acquired using an echo-planar imaging (EPI) gradient echo sequence covering the brain and cervical spinal (TR/TE = 1550/23 ms, axial FOV = 120 x 120 $mm^2$, flip angle = 70°, in-plane voxel resolution = 1.6 x 1.6 $mm^2$; slice thickness = 4 mm (no gap); iPAT acceleration factor PE = 2, iPAT acceleration factor slice = 3). A total of 69 axial slices were acquired covering the top of the head to approximately T1 vertebra. Only the slices containing the cervical spinal cord were included in further analyses. One resting state-run (*i.e.*, no explicit task) was acquired in total with an acquisition time of 10 minutes and 35 seconds each (230 volumes). Participants were instructed to relax watch the video and minimize swallowing and motion. Physiological recordings were acquired using a pulse sensor and a respiration belt (Siemens Physiology Monitoring Unit).

## Spinal cord analysis pipeline

We utilized bash programming, the FMRIB Software Library (FSL) (Smith et al., 2004), Spinal Cord Toolbox (SCT version 6.0) (De Leener et al., 2017), and in-house MATLAB programs to process the collected data. The full spinal cord processing pipeline will be publicly available at www.vahdatlab.org. The main steps of the spinal cord processing pipeline are shown in Fig. 1, including preprocessing, registration to the spinal cord template (PAM50 from SCT toolbox), and regression analysis, as described below.

## Preprocessing

Following removing of the first few volumes to reach a steady state condition (in our dataset, no volume was removed due to the inclusion of dummy scans during the acquisition), motion correction was performed on the EPI data in two steps (Fig. 1). First, a 3d linear rigid-body transformation was performed using mcflirt tool (FSL) on the entire brain and spinal cord FOV. Next, the EPI was divided into the spinal cord and brain FOVs. Slice-wise (2d) non-linear motion correction was then performed in a relatively large (23 voxels diameter) cylindrical mask of the spinal cord using the *sct_create_mask* (SCT). The performance of various motion correction methods (3d linear alone, slice-wise nonlinear alone, or both) was calculated and compared using two indices: i) DVARS that measures changes in BOLD signal intensity from one volume to the next (Eq. 1, (Afyouni and Nichols, 2018), and ii) tSNR (temporal signal-to-noise-ratio) which is the ratio of the mean by the standard deviation of the BOLD signal timeseries ($Y_t$) in each voxel (Eq. 2), *n* being the number of acquired volumes.

Equation 1 $\mathrm{DVARS} = \sqrt{\frac{1}{n}\sum_{t=1}^{n}\left(Y_t - Y_{t-1}\right)^2}$

Equation 2 $\mathrm{tSNR} = mean\left(Y_t\right)/std\left(Y_t\right)$

Following motion correction, gradient-echo field map correction was performed using FUGUE tool (FSL) to correct for geometrical distortion due to magnetic field inhomogeneities (Fig. 1). These artifacts are usually larger in the spinal cord compared to the brain due to the proximity to the thorax, vertebrae, and disks, and can cause a shift in the phase-encoding direction, which can be compensated at this step. Next, spatial smoothing was performed inside a mask of the spinal cord in a slice-wise fashion using *3dBlurToFWHM* command from AFNI (Cox, 1996). A kernel size of 3.2 mm (2 times the voxel size) was used for smoothing, although a 2.4 mm kernel size resulted in very similar task-based activation maps.

## Registration to template

The spinal cord registration pipeline is displayed in Fig. 2. Prior to registration, a spinal cord mask was generated for each of the T2w and T1w images using a deep learning segmentation algorithm as implemented in *sct_deepseg_sc* (SCT) with automatic centerline detection using the support vector machine and convolutional neural network options (Gros et al., 2019). Following visual inspection, manual corrections were carried out to address any minor inaccuracies in the spinal cord mask. Next, a centerline was drawn manually on the T2w and EPI images. To be consistent across participants, in the T2w image, the centerline in the dorsoventral direction was defined at a voxel location just posterior to the gray commissure, which was clearly visible in the acquired T2w image. For the EPI image, since the gray/white matter distinction is less clear, we used the calculated tSNR map to define the centerline. As shown in Fig. 3, the cord exhibits noticeably higher tSNR values compared to the surroundings, including the cerebrospinal fluid (CSF), blood vessels, and connective tissues. Hence, we identified the centerline at the midpoint of the area with the highest tSNR (yellow area in Fig. 3; when tSNR map is thresholded at 40, color-coded in red-yellow).

Registration to the template (PAM50) was performed in three steps (Fig. 2): 1) EPI image registration to the T2w space using slice-wise centerline images alignment, 2) T2w image registration to the T1w space through the cord segmentation images using *sct_register_multimodal* (SCT), and 3) T1w image registration to PAM50 through cord segmentation using *sct_register_to_template* (SCT). In detail, for step 1, EPI and T2w images were registered by performing slice-wise 2d in-plane translations (in the dorsoventral and mediolateral directions) to align their centerlines using flirt command (FSL) with the nearest-neighbor interpolation. For step 2, we manually identified the C1-T1 disc labels in both T1w and T2w images, and used their calculated cord segmentations images to compute a slice-wise rigid body transformation using *sct_register_multimodal* command (any mismatch in the rostrocaudal direction was first corrected by aligning T1w and T2w disk label locations). For step 3, the cord segmentation and disc labels in the T1w image were employed for registration to PAM50 through *sct_register_to_template* command, using the default options (including straightening, vertebral alignment, and non-linear slice-wise registration). Note that only step 1 was applied to the 4d EPI images. The steps 2 and 3 warping transformations were concatenated using *sct_concat_transfo*, and were later applied to the summary statistics images (regression coefficients and their variance images) using *sct_apply_transfo command*.

Subsequently, we compared the performance of the proposed registration method with the gold-standard spinal cord registration approach which only utilizes the T1W image (Landelle et al., 2023; Vahdat et al., 2015). This approach was implemented by calculating the transformations from the EPI to T1W image using *sct_register_multimodal*, and from T1W image to PAM50 as calculated above. The two transformations are then concatenated to register the EPI image directly to PAM50. To evaluate the registration performance, the binary cord segmentation mask in the EPI space was registered to PAM50 space using each method, and the result was compared to the binary template cord mask. Two indices were defined by calculating: i) the number of voxels in the template mask that are missed in the registered mask, normalized by the total number of template cord voxels, and ii) the overlap between the registered and the template cord masks, normalized by the total number of voxels in both masks.

## Regression analysis

We performed participant-level general linear model (GLM) analyses on the preprocessed EPI data in the T2w space in two steps. First, a GLM was constructed to model and remove the effects of three sets of confounds regressors, including: i) the mean CSF signal averaged in a surround mask generated by dilating the mask of spinal cord (*i.e.*, GM + WM), ii) 6 motion correction parameters (x, y, and z translations and rotations) derived from the linear motion correction step, and 2 slice-wise motion correction regressors generated by the non-linear motion correction step on the spinal cord FOV, and iii) 5 regressors, identified as confound, extracted from independent component analysis (ICA). The fastica algorithm (Hyvarinen, 1999) was used to decompose the preprocessed spinal cord EPI data (before spatial smoothing) within a large mask (including both the spinal cord and the surround mask) into 30 components (Vahdat et al., 2020). We calculated 3 indicators to systematically sort components based on their likelihood of being task-related, as opposed to those related to physiological/scanner noise: 1) spatial indicator: the ratio of significantly active ($p < 0.05$) voxels within the spinal cord mask to that within the surround mask, 2) frequency indicator: the ratio of the power of the component’s time-series frequency response around the task central frequency (in our block-design paradigm: 1 / (task period +

rest period) = 1/40 s = 0.025 Hz; 0.01 < *f* < 0.1 Hz may be used for resting-state data) to that outside the task-related frequency range with some margins (*f* < 0.025/1.5 and *f* > 0.025*1.5 Hz), and 3) temporal indicator: the Pearson correlation between the task timing signal (boxcar function in our task) convolved with a canonical hemodynamic response function (HRF) and the time-series of each component. A *confound index* was defined by multiplying the 3 indicators; the lower the *confound index*, the higher the likelihood that component represents a confound. The 5 components with the least *confound index* were selected, and their time series were included in the first GLM as confound.

Next the residual (clean) image from the first GLM was high pass filtered at 1/60 Hz. The timing of the grip force block design task was modeled as a regressor of interest, while motion outliers were modeled as confounds. The latter were calculated using *fsl_motion_outliers* command on the non-preprocessed spinal cord EPI data (Fig. 1). The summary statistics images (*Cope* and *VarCope* images in FSL) related to the regressor of interest were then registered to PAM50 space using the concatenated warping transformation calculated in the registration step.

We generated a set of individual-specific masks to specify low tSNR voxels for each participant as follows. For each EPI run, a tSNR *threshold* was calculated using Maximum a Posteriori (MAP) estimation of two Gaussian distributions on the calculated tSNR histogram data inside a large spinal mask (including both the cord and the surround binary masks; Fig. 3). A Gaussian mixture distribution model using two components was fitted to the tSNR histogram using MATLAB *fitgmdist* function. The *threshold* was defined as the MAP estimate of the optimal boundary, which is the intersection of the two Gaussian distributions (Fig. 3). Low tSNR voxels (tSNR < *threshold*) were identified for every EPI run and participant. A subject-specific low tSNR mask was then defined as the intersection of low tSNR voxels from all EPI runs for each participant (a total of 2 runs in our data). Note that the two Gaussian distributions represent, hypothetically, the spinal cord and the CSF/surround voxels, with high tSNR voxels expected to be located inside the spinal cord mask as shown in Fig. 3. Hence, the goal of this step is to calculate an optimal threshold for differentiating spinal cord and CSF/surround voxels and identify those voxels inside the spinal cord mask that, inconsistently, possess low tSNR due to severe distortion and signal loss in the EPI spinal data.

Finally, a group-level GLM model was constructed to average individual-level maps using a mixed-effect (FLAME 1 + 2) model as implemented in FSL. The individual-specific low tSNR masks modeling the low tSNR voxels in each participant were entered as confound (Fig. 1). First we generated a group-level spinal cord mask that contains voxels, in which the majority of participants showed an acceptable tSNR (*i.e.*, tSNR > *threshold*). This condition ensures that for every voxel in the group-level mask, high quality data from at least half of the participants were included for averaging, and, at the same time, limits the number of confound regressors to half the degrees of freedom in the group-level GLM. Hence, a total of up to *n* = (number of participants / 2) 4d voxel-wise regressors were included to account for and remove the effect of low tSNR voxels in every participants from group-level averaging. This procedure enabled us to only incorporate voxels with an acceptable tSNR in the group-level averaging, without the need to remove these voxels from the group-level mask/analysis. All group-level statistical maps were then corrected for multiple comparisons using Gaussian random field (GRF) theory, $Z > 2.7$, and cluster-level threshold $p < 0.05$.

## Brain fMRI processing

Brain image preprocessing was performed with the FSL software package, using the same pipeline as described previously (Vahdat et al., 2020, 2015). One participant was removed from brain analysis due to partial coverage of brain FOV. Preprocessing included the following steps: skull stripping (FSL, BET), motion correction (FSL, MCFLIRT), high-pass temporal filtering (1/60 Hz), GRE field map correction, spatial smoothing using an isotropic Gaussian kernel of 3.2 mm full width at half maximum. BBR method (FSL) was used to register the EPI data to the T1w anatomical image, followed by a nonlinear registration (FNIRT, FSL) from the T1w image to the MNI template. These two transformations were concatenated to register the summary statistics images from the participant level analysis in the EPI space to the MNI template. Participant-level GLM was constructed to identify brain areas activated during motor control task using a boxcar function modeling our block-design paradigm. The 6 motion correction parameters (x, y, and z translations and rotations) derived from the linear motion correction step were included as confound in this analysis. Furthermore, volumes detected as motion outliers (described earlier) were included as confound in the GLM. Next, a group-level GLM model was constructed to average participant-level maps using a mixed-effect (FLAME 1 + 2) model as implemented in FSL. All group-level statistical maps were then corrected for multiple comparisons using Gaussian random field (GRF) theory, $Z > 2.7$ (voxel-wise uncorrected threshold), and a cluster-wise corrected threshold at $p < 0.05$.

## Functional connectivity analysis

We performed region of interest (ROI)-based functional connectivity analysis to examine the communication between the spinal cord and brain BOLD time-series. We defined 11 *a-priori* selected ROIs in the brain shown to be part of the sensorimotor network using the MNI brain atlas and brainstem atlas (Cauzzo et al., 2022; Singh et al., 2021; Vahdat et al., 2020). The brain ROIs included: left precentral gyrus, left postcentral gyrus, supplementary motor area, left thalamus, right cerebellum lobule I-IV, right cerebellum lobule V-VI, left red nucleus, bilateral pontine reticular formation, bilateral medullary reticular formation. We also defined 2 ROIs in the spinal cord (right ventral horn C5-C8, right dorsal horn C5-C8 segmental levels). To restrict our analysis to subregions somatotopically related to the forelimb region, each ROI only included the most active voxels during handgrip force production by selecting voxels with the highest *Z*-score from the individual-level GLM analysis (top 10, 20, 30, or 40th percentile voxels within each ROI). We then calculated the average clean BOLD signal (the residual image from the first GLM) within each ROI at each percentile threshold. For each participant and run, the partial correlation (Marrelec et al., 2006; Vahdat et al., 2014) was calculated between each pair of the brain and spinal cord ROI's time-series (22 pairs), by accounting for and removing the effects of task presentation (task timing convolved with HRF). The average partial correlation across 4 percentile thresholds were calculated and reported as the functional connectivity (FC) for each ROI pair. For each participant, FC values between the two runs were averaged and Fisher transformation was applied to meet normal distribution assumption. Finally, *t*-statistics were performed to identify significant functional connections during the motor task between the brain and spial cord ROIs across participants (corrected for multiple comparisons using Bonferroni correction, $p < 0.05$).

## Resting-state analysis

**Denoising pipelines.** To investigate the impact of different sources of noise we compared the performance of different denoising methods. For each methods, the fMRI time-series was regressed with various variables of no-interest (confounds) using FSL's fMRI Expert Analysis Tool (FEAT). No filtering was applied at this step. All methods incorporated 8 motion-related regressors, 6 obtained from the linear motion correction step, and 2 slice-wise regressors from the SCT motion correction step, as described earlier. Next, we extracted the mean signal of the CSF in a surround mask generated by dilating the mask of the spinal cord. For each participant, the physiological noise modelling was calculated using the Tapas Physio toolbox (Kasper et al., 2017). We used the RETROspective Image CORrection (RETROICOR) procedure (Glover et al., 2000) to generate noise regressors from peripheral physiological recordings (heart rate and respiration). Specifically, we modeled, four cardiac, four respiratory, harmonics, and four multiplicative terms for the interactions between respiratory and cardiac noise (32 regressors in total, similar to (Brooks et al., 2008; Kaptan et al., 2023; Kong et al., 2012). To identify non-neural fluctuations, we extracted the first five principal components of the unfiltered and unsmoothed CSF signal (in the surround mask) in the participant's native space using the CompCor approach (Behzadi et al., 2007). Additional regressors, identified as confound, were extracted from ICA in a dilated mask of the spinal cord as described in the Regression analysis section (3.89 ± 1.66 components).

Finally, a specific set of regressors of no-interest was accounted for in separate GLMs, corresponding to different denoising models as follow:

1. "*MeanCSF*" (baseline parameters, mean CSF)
2. "*ICA*" (baseline parameters, mean CSF, ICA components)
3. "*CompCor*" (baseline parameters, mean CSF, 5 CompCor components)
4. "*Retroicor*" (baseline parameters, mean CSF, 32 Retroicor components)
5. "*ICA + CompCor*" (baseline parameters, mean CSF, ICA and 5 CompCor components)

**Variance of the model.** For each method, we calculated the voxel-wise explained variance of the model ($R^2$) as a performance metric to provide deeper insights into the impact of noise removal from various sources using the following equation:

Equation 3 $\mathrm{R}^2 = [var\,(Y_t) - var\,(\epsilon_t)]/var\,(Y_t)$

Where $var\,(Y_t)$ and $var\,(\epsilon_t)$ denote respectively the variances of the fMRI times-series and the residual signal (*res4d.nii* in FSL) at each voxel, after applying the regression model associated with each method. Next, the average $R^2$ was calculated inside the surround mask (*i.e.*, the CSF mask), as well as the spinal cord mask. The ratio between the $R^2$ of CSF and spinal cord ($R^2_{CSF}$ / $R^2_{cord}$) is reported to evaluate the model's performance. A good model for removing the physiological noise would account for more variance in the CSF, while for less variance related to neural activation in the spinal cord. Hence, the larger the $R^2_{CSF}$ / $R^2_{cord}$ ratio, the greater the capacity of the model in explaining the BOLD signal variability inside the CSF compared to the spinal cord, and the better the model in removing the physiological noise.

Being aware of the limitations of the $R^2$ as a quality check metric, such as its failure to account for each model's degree of freedom, we also calculated the adjusted variance of the model ($AdjR^2$) as follows:

Equation 4 $$\mathrm{AdjR}^2 = 1 - \frac{(1-\mathrm{R}^2)(N-1)}{N-P-1}$$

Where *N* is the number of fMRI image time points, and *P* corresponds to the number of regressors of no-interests (confounds) included in each model. Unlike the $R^2$, which measures the proportion of variance explained by the model, $AdjR^2$ takes into account the degree of freedom of the model. This implies that with an increase in the number of regressors (resulting in the degree of freedom increase), the $AdjR^2$ value will increase only if the additional regressors enhance the overall model performance. The $AdjR^2$ was extracted in the CSF mask. t-statistics were performed to compare the $R^2$ and $AjdR^2$ between each model, and differences were considered significant with a *p*-value < 0.005, which was adjusted for multiple comparisons using Bonferroni correction (0.05 /10 = 0.005).

**Resting-state functional connectivity.** To assess the impact of denoising methods on functional connectivity measures, we conducted ROI-based analyses on the residual (cleaned) images. We defined 4 ROIs in the spinal cord (right ventral horn C5-C8, right dorsal horn C5-C8, left ventral horn C5-C8, left dorsal horn C5-C8) and 1 ROI in the surround mask cord (CSF C5-C8). We extracted and band-pass filtered (0.01–0.17 Hz)(Barry et al., 2018a) the time series within each ROI and calculated the average signal. Next, for each participant, we computed the partial correlation between each pair of spinal cord ROIs while the CSF average signal was used as a covariate. The Fisher-transformed correlation coefficients were employed to establish functional connectivity between each ROI pair, specifically between ventral horns (Ventro-Ventral FC), dorsal horns (Dorso-Dorsal DC), ventro-dorsal right horns (Right FC) and ventro-dorsal left horns (Left FC). For each FC, t-statistics were performed to compare the functional connectivity between each model (corrected for multiple comparisons using Bonferroni correction 0.05 /10 = 0.005).

## Results

We collected simultaneous spinal cord-brain fMRI data, covering the whole brain and the entire cervical cord down to the first thoracic vertebra, using simultaneous multi-slice (SMS) EPI sequence, together with the application of neck and brachial plexus SatPad™ set, as described in the Methods section. This image acquisition protocol provided a reasonable spatial and temporal resolution (1.6x1.6 $mm^2$ in plane, TR: 1.85 ms), as well as a reasonable tSNR in the spinal cord (22.32 ± 0.86 mean ± sem; Fig. 3) of our group of participants. Furthermore, the low tSNR voxels were statistically excluded, per participant, from the group-level analysis. On average 76 ± 2.1 (mean ± sem) percent of the spinal cord voxels passed the MAP

threshold and were included in group-level analysis. The average tSNR in the voxels included in the group-level analysis was 25.40 ± 0.81 (mean ± sem) across participants (Fig. 3).

## Motion correction procedures

First, we assessed the efficiency of various motion correction procedures in the spinal cord using DVARS (Eq. 1) and the spinal cord tSNR metrics (Eq. 2). Optimal outcomes encompass reduced DVARS and enhanced spinal cord tSNR values. The performance of 3 methods were compared to the raw (no motion correction) data, including 3d linear motion correction on the entire brain and spinal cord FOV (SP + BR Flirt), slice-wise nonlinear correction on the spinal cord FOV (Slice-wise SCT), and the combined procedure (first performing 3d linear correction on the entire FOV, followed by slice-wise nonlinear correction on the spinal cord slices) as implemented in FASB. As shown in Fig. 4, the combined procedure outperforms the other methods in terms of DVARS and tSNR (repeated-measures ANOVA, corrected for multiple comparisons using Bonferroni correction, **DVARS**: $F(3,42) = 14.3$, $p$ (FASB vs Raw) < 0.005, $p$ (FASB vs Flirt) < 0.05, $p$ (FASB vs SCT) < 0.05, $p$ (SCT vs Flirt) > 0.05; **Spinal cord tSNR**: $F(3,42) = 23.5$, $p$ (FASB vs Raw) < 0.001, $p$ (FASB vs Flirt) < 0.01, $p$ (FASB vs SCT) < 0.01, $p$ (SCT vs Flirt) > 0.05). This analysis revealed that combining both 3d linear and slice-wise non-linear motion correction procedures yields reduced temporal variability and enhanced temporal SNR in the spinal cord.

## Registration procedures

Next, we compared the performance of various registration methods to align the EPI spinal cord image to a template (PAM50). As outcome measures, we calculated percentage error and overlap between the registered binary EPI spinal cord mask and the PAM50 spinal cord segmentation, as reported in Methods. Smaller *Error* and larger *Overlap* percentage values are desirable. As described in the Methods, the performances of 3 procedures are compared, including registration using T1W image as the intermediate image (T1W-based), GRE field map correction together with T1W-based registration (GRE + T1W-based), and FASB registration procedure as summarized in Fig. 2. As shown in Fig. 5, the FASB registration procedure outperforms the other registration methods (which do not include T2w image) in terms of both *Error* and *Overlap* for the spinal cord masks (repeated-measures ANOVA, corrected for multiple comparisons using Bonferroni correction, **Error**: $F(2,28) = 37$, $p$ (FASB vs T1W) < 0.001, $p$ (FASB vs GRE + T1W) < 0.001, $p$ (T1W vs GRE + T1W) > 0.05; **Overlap**: $F(2,28) = 11.5$, $p$ (FASB vs T1W) < 0.001, $p$ (FASB vs GRE + T1W) < 0.05, $p$ (T1W vs GRE + T1W) > 0.05). This analysis shows that performing the additional slice-wise centerline alignment between EPI and T2w images, as proposed in FASB, significantly improves the overall accuracy of the spinal cord registration to the template space (from 78–84% overlap).

## Physiological noise reduction procedures

Next, we compared the performance of different physiological noise removal models on the collected resting-state fMRI data. We compared 5 models (see Methods for details): *"meanCSF"* (the baseline model for removing motion correction parameters and the CSF average), *"ICA"* (as implemented in FASB), *"CompCor"* (a PCA-based model), *"Retroicor"* (based on the recorded cardiac and respiratory signals), and

*“ICA + CompCor”* (combined *“ICA”* and *“CompCor”* models). First, we compared the ratio between the $R^2$ of CSF and spinal cord ($R^2_{CSF}$ / $R^2_{cord}$) between all denoising models to evaluate model performances (Fig. 6A). A higher value indicates that the variance of the CSF is explained more substantially by the model relative the variance of the cord. Results showed a significant increase across most of the denoising models compared to the baseline model “*meanCSF*” ("*ICA*” *vs*. “*meanCSF*: t(17) = 4.67, p = 0.0002; "*CompCor*” *vs*. “*meanCSF”*: t(17) = 4.87, p = 0.0001; "*ICA + CompCor*” *vs*. “*meanCSF*: t(17) = 4.34, p = 0.0004), with the exception of the “*Retroico*r” model where no significant difference was observed with the “*meanCSF*” model (t(17) = *1.12, p < 0.27*). In addition, “*Retroico*r” model showed a lower ratio than “CompCor” (t(17)= -5.38, p < 0.0001), “ICA” (t(17)= -4.02, p = 0.0009) and “*ICA + CompCor*” (t(17) = 5.97, p < 0.0001) models. *“CompCor”* model had significant higher ratio than *“ICA”* (*"CompCor*” *vs*. “*ICA” (t(17) = 3.45, p = 0.003)*, while no significant differences, after the Bonferroni correction, were observed between the *“ICA*” and “*ICA + CompCor”* (t(17) = 3.03, p = 0.007*)*, or "*CompCor*” and “*ICA + CompCor” (t(17) = 2.60, p = 0.018*).

To account for the degrees of freedom of each model (depending on the number of confound regressors), we also calculated the adjusted $R^2$ (Adj$R^2$; Eq. 4) inside the CSF mask. Adding new confound regressors does increase the Adj$R^2$ value only if the additional regressors enhance the overall model performance. As shown in Fig. 6B, all models have higher Adj$R^2$ than the baseline model (*“ICA*” *vs*. “*meanCSF*”: t(17) = 12.89, p < 0.0001; *“CompCor*” *vs*. “*meanCSF*” : t(17) = 24.25, p < 0.0001; *“Retroicor*” *vs*. “*meanCSF”: t(17) = 7.01, p < 0.0001; “ICA + CompCor*” *vs*. “*meanCSF*”: t(17) = 24.38, p < 0.0001). “*Retroico*r” model showed lower Adj$R^2$ than “*CompCor*” (t(17)= -20.40, p = 0.0001), “ICA” (t(17)= -7.07, p < 0.0001) and “*ICA + CompCor”* (t(17)= -19.45, p < 0.0001) models. *“ICA + CompCor”* model had significantly higher Adj$R^2$ than *“ICA”* (t(17) = 20.40, p < 0.0001) and *“CompCor”* models (t(17) = 9.33, p < 0001). Finally “*CompCor”* model showed significantly higher Adj$R^2$ than *“ICA”* model (t(17) = 10.35, p < 0001).

## Assessing the impact of denoising procedures on functional connectivity

Next to assess the impact of denoising methods on functional connectivity (FC) measures, we conducted ROI-based analyses (see Methods). As shown in Fig. 6C, no significant differences in FC between the different denoising models were observed associated with ipsilateral functional connectivity measures (ventro-dorsal right, and ventro-dorsal left). *“ICA + CompCor”* model led to significantly reduced functional connectivity between the left and right ventral horns compared to most of the other models (“*meanCSF*”: t(17)= -3.86, p = 0.001; “*ICA*”: t(17)= -3.80; p = 0.001; “*Retroicor*”: t(17)= -4.36, p = 0004) and a trend toward significant when compared to the “*CompCor*” model (“*ICA + CompCor*” *vs*. “*CompCor*” t(17) = 3.1, p = 0.006). Similarly, the FC between the left and right dorsal horns was significantly lower for the “*ICA + CompCor*” model compared to the others (“*meanCSF*”: t(17)= -5.59, p < 0.0001, “*ICA*”: t(17)= -4.02, p = 0.0008, “*Compcor*”: t(17)= -3.62, p = 0.002, “*Retroicor*”: t(17)= -6.19, p < 0.0001). The FC was also found lower with “ICA” model compared to “*meanCSF*” (t(17):-4.46, p = 0.0003) and “*Retroicor*” (t(17)= -4.56, p = 0.0002) models. In sum, ventro-ventral and dorso-dorsal connections showed reduction in functional connectivity in the more complex model (“*ICA + CompCor*”) compared to the others. Interestingly for the weaker FC observed within horns (ventro-dorsal FC), the various denoising models did not significantly impact the FC.

## Task-related results

Figure 7 shows activation clusters associated with the motor task performance in the brain and brainstem. At the brain level, all of the anticipated sensorimotor cortical and subcortical areas related to unilateral hand movement (Vahdat et al., 2017, 2015, 2011) showed significant activation, including the contralateral primary motor cortex (M1), dorsal premotor cortex, primary and secondary somatosensory cortices (SI, SII), thalamus (ventral lateral and pulvinar nuclei), putamen, ipsilateral cerebellar cortex (lobules I-IV, and V-VI), bilateral posterior parietal cortex (PPC), and supplementary motor area (SMA) (corrected for multiple comparisons using GRF, $Z > 2.7$, $p < 0.5$, Fig. 7A). Interestingly, our high in-plane spatial resolution (1.6×1.6 $mm^2$) allowed us to detect and delineate task-related brainstem activations, including bilateral pontine reticular formation (PRF), medullary reticular formation (MRF), and red nucleus (RN) (Fig. 7B). Consistent with the laterality of descending projections from the reticular formation and RN (Davidson et al., 2007; Lemon, 2008; Soteropoulos et al., 2012), task-related activation was greater in the ipsilateral MRF and PRF, and the contralateral RN. These results show that the acquired EPI brain images in our simultaneous protocol not only provide enough power to replicate previous task-related brain networks, but also offer detection and delineation of activity in important brainstem nuclei.

Next, we also compared task-related activation maps in the spinal cord using different approaches. Four methods were implemented and compared as described in the Methods section: i) regular group-level regression, without the inclusion of ICA confounds (NoICA-NotSNR), ii) regular group-level regression, with the inclusion of ICA confounds (ICA-NotSNR), iii) group-level regression by inclusion of voxel-wise low-tSNR confounds, but without ICA confounds (NoICA-tSNR), and iv) group-level regression by inclusion of voxel-wise low-tSNR confounds, as well as ICA confounds (ICA-tSNR, implemented in FASB). As shown in Fig. 8, all 4 methods resulted in significant activation clusters at the C5-C8 cervical segmental levels (corrected for multiple comparisons using GRF, $Z > 2.7$, $p < 0.05$). Compared to the other methods, FASB (ICA-tSNR) resulted in the largest number of activation clusters (4 clusters, including C5, C6, C7, and C8) and detected activation across all related cervical levels for the control hand/wrist muscles (Landelle et al., 2021). Importantly, the largest peaks of activation were unequivocally located in the ipsilateral ventral horn, where the hand/wrist motoneurons are located. The activation at the C6-C7 level was largely restricted to the ventral horn, while it included the dorsal horn at the C8 segmental level. These results show that the acquisition/analysis pipeline proposed in FASB can differentiate task-related spinal cord activation based on myotome/dermatome (spinal level), side (left/right), and sensory-motor (dorsal/ventral) characteristics.

To evaluate the connectivity related to the ascending and descending projections, we next investigated the partial correlation between *a-priori* selected set of ROIs in the brain and spinal cord, when the effects of task paradigm are removed from this relationship (see Methods for details). The right (ipsilateral)

ventral horn at C5-C8 cervical level showed significant functional connectivity to the left postcentral gyrus, SMA, thalamus, and right medullary reticular formation and cerebellum lobule V-VI (Fig. 9; $p < 0.05$). Also, the right dorsal horn at C5-C8 cervical level showed significant functional connectivity to the left precentral and postcentral gyrus, SMA, thalamus, and right cerebellum lobule V-VI. These results demonstrate that significant task-related functional connectivity between the brain and spinal cord ROIs can be identified using FASB, consistent with the neuroanatomy of well-established descending and ascending projections in humans (Landelle et al., 2021; Lemon, 2008).

## Discussion

We developed and validated an integrated processing pipeline for functional analysis of simultaneous spinal cord-brain EPI data (FASB), together with recommendations on collecting appropriate anatomical and functional data for that pipeline. The proposed EPI acquisition allowed us to acquire spinal cord images using a Siemens 3T MRI scanner, with low distortion due to magnetic susceptibility artifacts, and with high SNR (mean temporal SNR > 20 in the cord), while having a relatively high spatial resolution at the spinal cord level (1.6 mm in-plane), and a reasonable acquisition time (TR = 1.85 s). The novel aspects of the FASB pipeline are threefold (Fig. 1): 1) Improved motion correction of spinal cord fMRI volumes using both 3d linear and slice-wise non-linear transformations, 2) Improved spinal cord EPI registration to the template space (PAM50) using two intermediate anatomical images (T2w and T1w), and slice-wise centerline alignment to T2w image guided by the tSNR map of the EPI data (Fig. 2), and 3) Improved detection power of the group-level analysis by removing the effects of low tSNR voxels in each participant, typically at the disk level, based on the MAP estimate of the tSNR threshold between the spinal cord and CSF/surround voxels (Fig. 3).

The simultaneous acquisition of brain and spinal cord enabled us to use spatial information from both FOVs for motion correction of the spinal cord EPI volumes. Based on our results reported in Fig. 4, performing a linear transformation on the entire brain and spinal cord FOVs, followed by slice-wise non-linear correction at each spinal cord slice significantly improves the overall motion correction performance. This is likely due to the 3d linear correction step, which effectively addresses motion correction along the rostro-caudal direction, where the slice-wise correction step is insensitive.

Furthermore, FASB registration of the EPI to the template space significantly improves the spinal cord registration compared to current spinal registration approaches (Landelle et al., 2023; Vahdat et al., 2015). Spinal cord EPI images are often spatially distorted at the disk level, and performing a nonlinear transformation generates non-optimal twisted warping fields. Magnetic susceptibility artifacts also result in signal loss in the distorted voxels, which makes the inclusion of these voxels in further analyses even more problematic. Hence, we proposed performing a simple slice-wise centerline alignment to match the centerlines of the EPI and T2w images. This is possible because we acquire a T2w image at the same angle/slice thickness as the EPI image. Moreover, to standardize the selection of centerline specifically in the distorted slices, we propose to select the EPI centerline based on the estimated tSNR map (Fig. 3). Lastly, we account for and remove the effects of distorted slices later by including the signal of participant-specific low tSNR voxels as confound in the group-level regression analysis. Although the T2w medic image is one of the requirements for our registration pipeline, because of its sharp contrast for discerning the gray and white matter, it can also provide useful morphometric information that may be of interest in different clinical populations.

For systematic selection of the tSNR threshold between the spinal cord and CSF/surround voxels, we propose to use the optimal boundary (MAP estimate) between two Gaussian distributions, fitted on the tSNR histogram. This makes our threshold selection procedure unbiased and dependent upon the quality of the acquired functional data. In our collected EPI data, the average tSNR threshold was 12.96 (± 0.40 sem). This allowed us to identify the "low tSNR voxels" (*i.e.*, those inside the spinal cord mask whose tSNR was lower than the MAP estimate), and remove their effect at the group-level analysis by the inclusion of participant-specific voxel-wise regressors. To the best of our knowledge, our proposed method provides the only available solution to account for and remove the effects of low tSNR voxels from the group-level results, which is particularly critical in spinal cord fMRI due to large magnetic susceptibility artifacts at the spinal cord level.

For removal of the scanner noise/physiological artifacts, which are particularly intensified in spinal cord fMRI, we propose to use ICA together with automatic selection of confound components based on their spatial, frequency, and temporal characteristics. Our results show that the performance of ICA and *RETROICOR* (Glover et al., 2000) (based on recorded cardiac and respiratory rates) methods are comparable in terms of reducing the variability attributed to noise (Fig. 6A-B). Combining the confound regressors extracted using *CompCor* method (Behzadi et al., 2007) (a PCA-based approach) and ICA improves the noise removal performance as evaluated by the explained variance in CSF. However, it should be noted that CSF outcome measures are generally biased toward *CompCor*, as this method performs PCA inside the CSF mask. Importantly, the analysis of functional connectivity between various spinal cord quadrants (Fig. 6C) revealed that the various denoising models (*CompCor*, ICA, *RETROICOR*) did not significantly impact the calculated functional connectivity results. Hence, we included ICA as the method of choice in FASB, due to the ease of acquisition (no need for cardiac and respiratory recordings). However, note that our ICA confound selection procedure is particularly suited for task-based fMRI, in which the frequency and temporal criteria can be defined based on the task timing. In resting-state paradigms selection of confound components can still be achieved (e.g., more active voxels inside the CSF than the spinal cord mask, and larger power outside the neural-related frequency range [0.01 Hz, 0.17 Hz] (Barry et al., 2018a) than within this range), though it is not as straightforward as in the bask-based design. Hence, for resting-state analysis, a combination of ICA, *CompCor* or *RETROICOR* methods may be considered.

For validation purposes, we examined the activation maps in the brain and spinal cord using FASB during the performance of a hand force control task. The force control task was selected due to 3 main advantages compared to similar motor tasks performed in the scanner: 1) The task is isometric, so there is no difference in movement kinematics across participants, 2) The produced force is normalized across individuals using their MVC, and 3) The isometric task minimizes motion artifacts, which are critical for spinal cord imaging at the neck level. The activated cortical and subcortical areas during this task are consistent with our previous fMRI studies using hand movements (Braaß et al., 2023; Vahdat et al., 2017, 2015, 2011), including contralateral M1, S1, premotor cortex, SII, PPC, thalamus, striatum, SMA, and ipsilateral cerebellum (Fig. 7). Importantly, we found significant task-related activations at the C5-C8 cervical level, mainly at the ipsilateral ventral horn (Fig. 8), consistent with the known neuroanatomical location of the finger/wrist motoneurons. Compared to our previous simultaneous spinal cord-brain fMRI studies (Khatibi et al., 2022; Kinany et al., 2023; Vahdat et al., 2020, 2015), in which inclusion of more than 25 participants were typically recruited to detect significant spinal cord activations, analysis of only 15 participants using the FASB pipeline resulted in significant focal spinal cord activation.

Importantly, simultaneous spinal cord-brain fMRI enables investigation of functional connectivity related to the descending and ascending pathways (Landelle et al., 2021; Vahdat et al., 2020). Our ROI-based analyses demonstrated significant functional connectivity during task performance between cortical, thalamic, cerebellar, and bulbar regions with the cervical ventral horn, and between cortical, thalamic and cerebellar regions with the cervical dorsal horn (Fig. 9). Interestingly, we found significant functional connectivity between the ipsilateral medullary reticular formation and the cervical ventral horn, consistent with the involvement of the reticulospinal pathway in hand motor control (Baker, 2011; Soteropoulos et al., 2012). Since the goal of the current study was to validate FASB pipeline for spinal cord fMRI processing, we performed a simple ROI-based partial correlation analysis to evaluate spinal cord-brain functional connectivity. Partial correlation analysis can account for and remove the effects of task presentation from the BOLD signal time-series, so that their correlation does not simply represent co-activation of areas during task periods as compared to rest (Marrelec et al., 2006). Other methods based on ICA (Vahdat et al., 2020, 2012), or BASCO (Rissman et al., 2004) (Beta series correlation, particularly suited for an event-related task design) may be used to assess functional connectivity between the brain and spinal cord during task or resting-state conditions. Although our results show that a simple ROI-based correlation analysis may be used to identify significant functional connections in line with the pattern of major descending and ascending projections in humans, future investigations are needed to evaluate the performance of various brain-spinal cord functional connectivity methods.

Furthermore, the improved in-plane spatial resolution at the brain level enabled us to detect significant activation clusters in important brainstem nuclei, including the reticular formation (MRF, and PRF), and red nucleus. Importantly, we detected bilateral activation clusters in PRF and MRF, with stronger activation on the ipsilateral side (Fig. 7), consistent with the observed pattern of reticulospinal projections targeting forelimb muscles in primates (Sakai et al., 2009; Soteropoulos et al., 2012). The contribution of various brainstem nuclei in the reticular formation, including the lateral rostral medulla (Arber and Costa, 2022; Ruder et al., 2021; Ruder and Arber, 2019), and caudal medulla (Esposito et al., 2014) (corresponding to MRF in humans) in forelimb motor control has been recently the topic of intense investigations in rodents. We hope that FASB acquisition/analysis pipeline provides a standard method for examination of task-related activation and functional interaction across cortical, subcortical, brainstem, and spinal cord levels, and enables testing/translation of preclinical findings to humans and clinical populations.

## Declarations

## Funding:

Research reported in this study was supported by NIH NINDS under Award Number R01NS131227 (PI: Vahdat), and Florida Department of Health James and Esther King Biomedical Research, Award Number 23K07 (PI: Vahdat). This work was supported in part by an NIH award, S10 OD021726, for High End Instrumentation. JD was supported by the Courtois Foundation, https://www.canadahelps.org/en/charities/fondation-courtois. C.L was supported by Fonds de Recherche du Québec—Santé (FRQ-S) and the Canadian Institutes of Health Research (CIHR).

## Author Contribution

S.V. designed the experiments, optimized the pipeline, and wrote the codes. S.V., F.B., C.L. collected the data, performed data analysis and generated the figures. S.V., O.L., B.D., J.D., F.B., and C.L wrote and revised the manuscript. All authors reviewed the manuscript.

2594(200007)44:1<162::AID-MRM23>3.0.CO;2-E

22. Gros, C., De Leener, B., Badji, A., Maranzano, J., Eden, D., Dupont, S.M., Talbott, J., Zhuoquiong, R., Liu, Y., Granberg, T., Ouellette, R., Tachibana, Y., Hori, M., Kamiya, K., Chougar, L., Stawiarz, L., Hillert, J., Bannier, E., Kerbrat, A., Edan, G., Labauge, P., Callot, V., Pelletier, J., Audoin, B., Rasoanandrianina, H., Brisset, J.C., Valsasina, P., Rocca, M.A., Filippi, M., Bakshi, R., Tauhid, S., Prados, F., Yiannakas, M., Kearney, H., Ciccarelli, O., Smith, S., Treaba, C.A., Mainero, C., Lefeuvre, J., Reich, D.S., Nair, G., Auclair, V., McLaren, D.G., Martin, A.R., Fehlings, M.G., Vahdat, S., Khatibi, A., Doyon, J., Shepherd, T., Charlson, E., Narayanan, S., Cohen-Adad, J., 2019. Automatic segmentation of the spinal cord and intramedullary multiple sclerosis lesions with convolutional neural networks. Neuroimage 184, 901–915. https://doi.org/10.1016/j.neuroimage.2018.09.081
23. Hyvarinen, A., 1999. Fast and robust fixed-point algorithms for independent component analysis. IEEE Trans Neural Netw 10, 626–634. https://doi.org/10.1109/72.761722
24. Islam, H., Law, C.S.W., Weber, K.A., Mackey, S.C., Glover, G.H., 2019. Dynamic per slice shimming for simultaneous brain and spinal cord fMRI. Magn. Reson. Med. 81, 825–838. https://doi.org/10.1002/MRM.27388
25. Kaptan, M., Horn, U., Vannesjo, S.J., Mildner, T., Weiskopf, N., Finsterbusch, J., Brooks, J.C.W., Eippert, F., 2023. Reliability of resting-state functional connectivity in the human spinal cord: Assessing the impact of distinct noise sources. Neuroimage 275. https://doi.org/10.1016/J.NEUROIMAGE.2023.120152
26. Kasper, L., Bollmann, S., Diaconescu, A.O., Hutton, C., Heinzle, J., Iglesias, S., Hauser, T.U., Sebold, M., Manjaly, Z.M., Pruessmann, K.P., Stephan, K.E., 2017. The PhysIO Toolbox for Modeling Physiological Noise in fMRI Data. J. Neurosci. Methods 276, 56–72. https://doi.org/10.1016/J.JNEUMETH.2016.10.019
27. Khatibi, A., Vahdat, S., Lungu, O., Finsterbusch, J., Büchel, C., Cohen-Adad, J., Marchand-Pauvert, V., Doyon, J., 2022. Brain-spinal cord interaction in long-term motor sequence learning in human: An fMRI study. Neuroimage 253, 119111. https://doi.org/10.1016/J.NEUROIMAGE.2022.119111
28. Kinany, N., Khatibi, A., Lungu, O., Finsterbusch, J., Büchel, C., Marchand-Pauvert, V., Van De Ville, D., Vahdat, S., Doyon, J., 2023. Decoding cerebro-spinal signatures of human behavior: Application to motor sequence learning. Neuroimage 275. https://doi.org/10.1016/J.NEUROIMAGE.2023.120174
29. Kinany, N., Pirondini, E., Micera, S., Van De Ville, D., 2022. Spinal Cord fMRI: A New Window into the Central Nervous System. Neuroscientist. https://doi.org/10.1177/10738584221101827
30. Kong, Y., Jenkinson, M., Andersson, J., Tracey, I., Brooks, J.C.W., 2012. Assessment of physiological noise modelling methods for functional imaging of the spinal cord. Neuroimage 60, 1538–1549. https://doi.org/10.1016/j.neuroimage.2011.11.077
31. Landelle, C., Dahlberg, L.S., Lungu, O., Misic, B., De Leener, B., Doyon, J., 2023. Altered Spinal Cord Functional Connectivity Associated with Parkinson’s Disease Progression. Mov. Disord. 38, 636–645. https://doi.org/10.1002/MDS.29354

32. Landelle, C., Lungu, O., Vahdat, S., Kavounoudias, A., Marchand-Pauvert, V., De Leener, B., Doyon, J., 2021. Investigating the human spinal sensorimotor pathways through functional magnetic resonance imaging. Neuroimage 245. https://doi.org/10.1016/J.NEUROIMAGE.2021.118684
33. Lemon, R.N., 2008. Descending pathways in motor control. Annu Rev Neurosci 31, 195–218. https://doi.org/10.1146/annurev.neuro.31.060407.125547
34. Marrelec, G., Krainik, A., Duffau, H., Pelegrini-Issac, M., Lehericy, S., Doyon, J., Benali, H., 2006. Partial correlation for functional brain interactivity investigation in functional MRI. Neuroimage 32, 228–237. https://doi.org/10.1016/j.neuroimage.2005.12.057
35. Pierrot-Deseilligny, E., Burke, D., 2012. The circuitry of the human spinal cord: Spinal and corticospinal mechanisms of movement, The Circuitry of the Human Spinal Cord: Spinal and Corticospinal Mechanisms of Movement. https://doi.org/10.1017/CBO9781139026727
36. Raudino, F., Leva, S., 2012. Involvement of the spinal cord in Parkinson’s disease. Int. J. Neurosci. 122, 1–8. https://doi.org/10.3109/00207454.2011.613551
37. Rissman, J., Gazzaley, A., D’Esposito, M., 2004. Measuring functional connectivity during distinct stages of a cognitive task. Neuroimage 23, 752–763. https://doi.org/10.1016/J.NEUROIMAGE.2004.06.035
38. Ruder, L., Arber, S., 2019. Brainstem Circuits Controlling Action Diversification. Annu. Rev. Neurosci. 42, 485–504. https://doi.org/10.1146/ANNUREV-NEURO-070918-050201
39. Ruder, L., Schina, R., Kanodia, H., Valencia-Garcia, S., Pivetta, C., Arber, S., 2021. A functional map for diverse forelimb actions within brainstem circuitry. Nature 590, 445–450. https://doi.org/10.1038/S41586-020-03080-Z
40. Sakai, S.T., Davidson, A.G., Buford, J.A., 2009. Reticulospinal neurons in the pontomedullary reticular formation of the monkey (Macaca fascicularis). Neuroscience 163, 1158–1170. https://doi.org/10.1016/J.NEUROSCIENCE.2009.07.036
41. Singh, K., García-Gomar, M.G., Bianciardi, M., 2021. Probabilistic Atlas of the Mesencephalic Reticular Formation, Isthmic Reticular Formation, Microcellular Tegmental Nucleus, Ventral Tegmental Area Nucleus Complex, and Caudal-Rostral Linear Raphe Nucleus Complex in Living Humans from 7 Tesla Magnetic Resonance Imaging. Brain Connect. 11, 613–623. https://doi.org/10.1089/BRAIN.2020.0975
42. Smith, S.M., Jenkinson, M., Woolrich, M.W., Beckmann, C.F., Behrens, T.E., Johansen-Berg, H., Bannister, P.R., De Luca, M., Drobnjak, I., Flitney, D.E., Niazy, R.K., Saunders, J., Vickers, J., Zhang, Y., De Stefano, N., Brady, J.M., Matthews, P.M., 2004. Advances in functional and structural MR image analysis and implementation as FSL. Neuroimage 23 Suppl 1, S208-19. https://doi.org/S1053-8119(04)00393-3 [pii]10.1016/j.neuroimage.2004.07.051
43. Soteropoulos, D.S., Williams, E.R., Baker, S.N., 2012. Cells in the monkey ponto-medullary reticular formation modulate their activity with slow finger movements. J. Physiol. 590, 4011–4027. https://doi.org/10.1113/jphysiol.2011.225169

44. Tinnermann, A., Büchel, C., Cohen-Adad, J., 2021. Cortico-spinal imaging to study pain. Neuroimage. https://doi.org/10.1016/j.neuroimage.2020.117439
45. Vahdat, S., Darainy, M., Milner, T.E., Ostry, D.J., 2011. Functionally specific changes in resting-state sensorimotor networks after motor learning. J Neurosci 31, 16907–16915. https://doi.org/10.1523/JNEUROSCI.2737-11.2011
46. Vahdat, S., Darainy, M., Ostry, D.J., 2014. Structure of Plasticity in Human Sensory and Motor Networks Due to Perceptual Learning. J. Neurosci. 34, 2451–2463. https://doi.org/10.1523/JNEUROSCI.4291-13.2014
47. Vahdat, S., Fogel, S., Benali, H., Doyon, J., 2017. Network-wide reorganization of procedural memory during NREM sleep revealed by fMRI. Elife 6, e24987. https://doi.org/10.7554/eLife.24987
48. Vahdat, S., Khatibi, A., Lungu, O., Finsterbusch, J., Büchel, C., Cohen-Adad, J., Marchand-Pauvert, V., Doyon, J., 2020. Resting-state brain and spinal cord networks in humans are functionally integrated. PLoS Biol. 18(7): e30. https://doi.org/10.1371/journal.pbio.3000789
49. Vahdat, S., Lungu, O., Cohen-Adad, J., Marchand-Pauvert, V., Benali, H., Doyon, J., 2015. Simultaneous brain–cervical cord fMRI reveals intrinsic spinal cord plasticity during motor sequence learning. PLoS Biol. 13, 1–25. https://doi.org/10.1371/journal.pbio.1002186
50. Vahdat, S., Maneshi, M., Grova, C., Gotman, J., Milner, T.E., 2012. Shared and specific independent components analysis for between-group comparison. Neural Comput. 24, 3052–3090. https://doi.org/10.1162/NECO_a_00355
51. Vaillancourt, D.E., Thulborn, K.R., Corcos, D.M., 2003. Neural Basis for the Processes That Underlie Visually Guided and Internally Guided Force Control in Humans. J. Neurophysiol. 90, 3330–3340. https://doi.org/10.1152/jn.00394.2003
52. Wahl, A.S., Schwab, M.E., 2014. Finding an optimal rehabilitation paradigm after stroke: Enhancing fiber growth and training of the brain at the right moment. Front. Hum. Neurosci. 8, 381. https://doi.org/10.3389/FNHUM.2014.00381/BIBTEX

## Figures

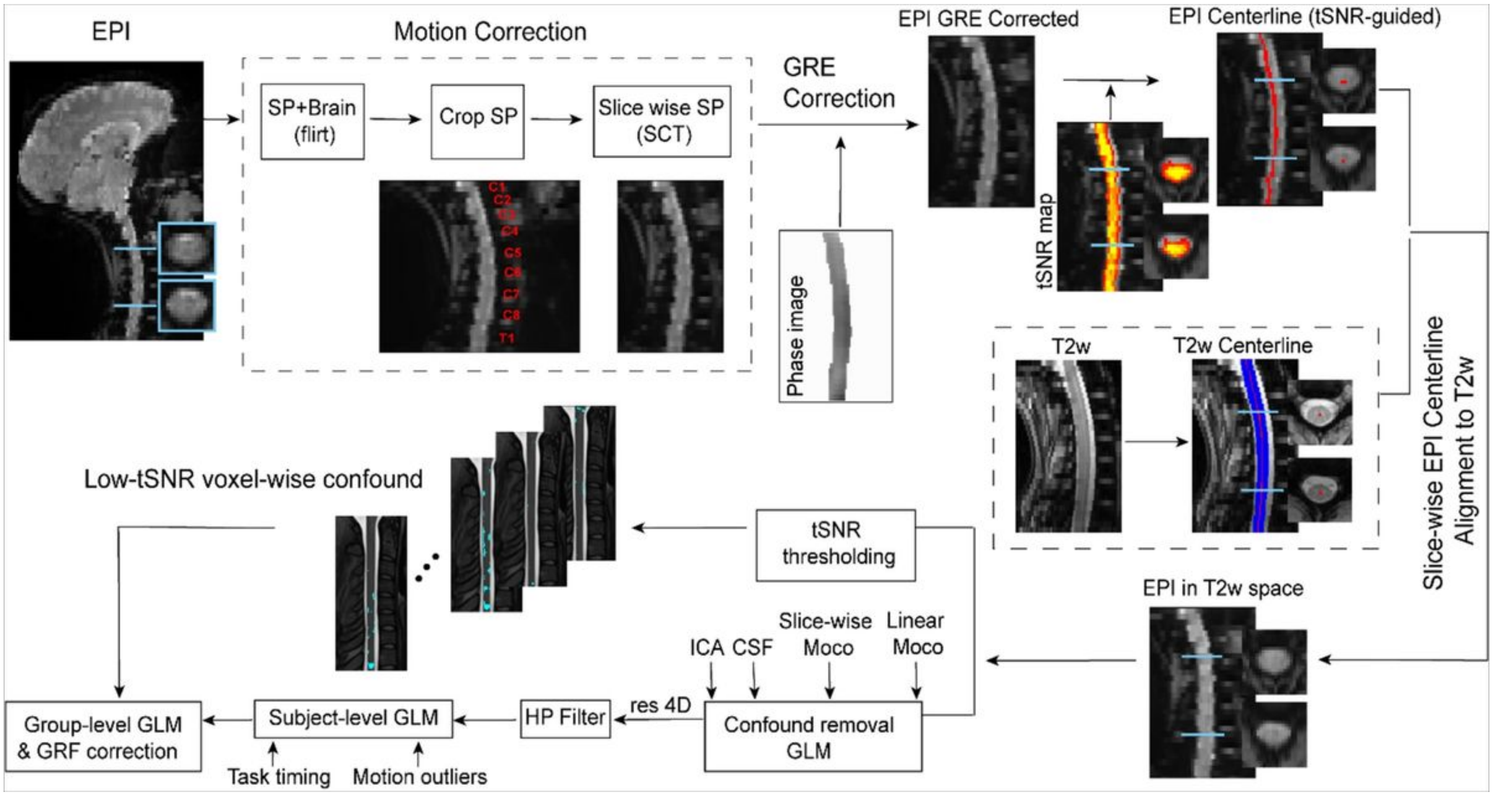

### Figure 1

**FASB EPI analysis pipeline.** The main steps for analysis of EPI spinal cord data include: 2-step motion correction, GRE field map correction, slice-wise centerline alignment to T2w image using tSNR map as a guide, MAP estimation of tSNR threshold, spatial smoothing, noise removal by regressing out motion correction parameters, average CSF signal, and ICA-based confounds, high-pass temporal filtering, individual-level regression using task timing as the regressor of interest and motion outliers, registration of the summary statistics images to the spinal cord template, group-level regression by inclusion of individual-specific voxel-wise low-tSNR confounds, and correction for multiple comparisons using GRF.

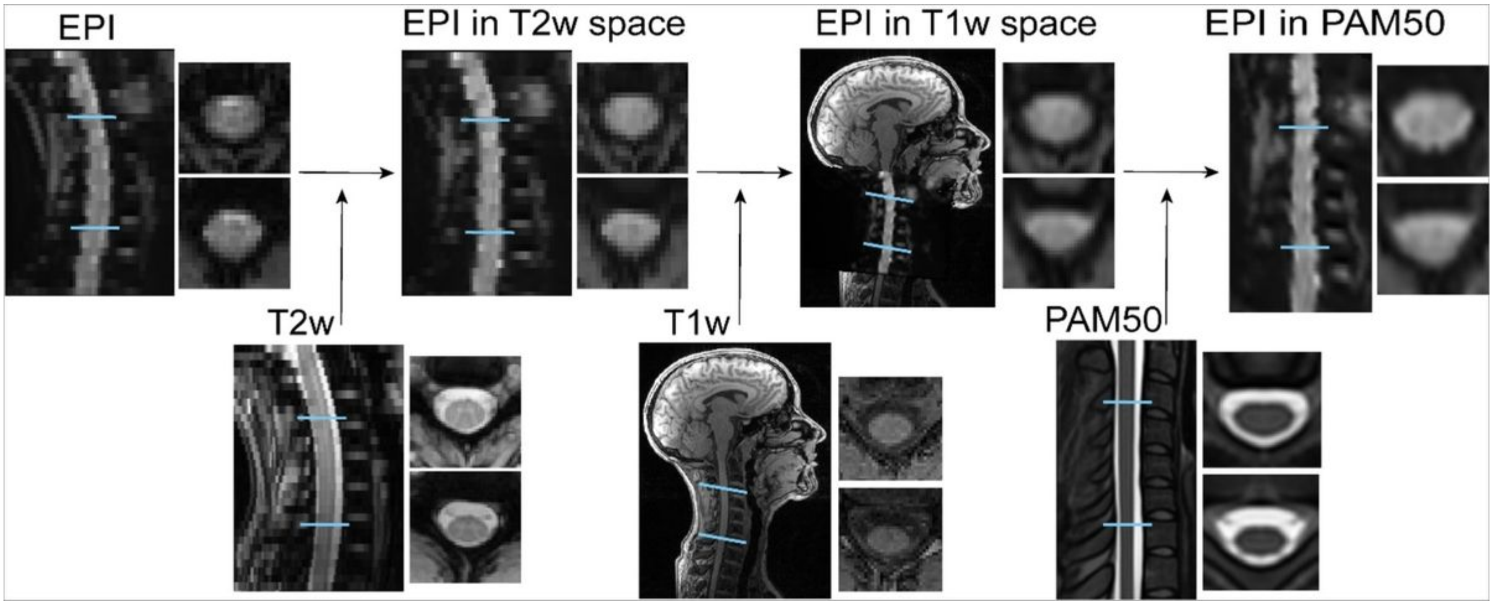

**Figure 2**

**FASB registration pipeline.** EPI spinal cord images are registered to the spinal cord template (PAM50) in 3 steps: 1) the reference EPI volume is registered to the T2w image using slice-wise centerline alignment, 2) T2w image is registered to T1w image via a slice-wise rigid body transformation using *sct_register_multimodal* (SCT), and 3) T1w image is registered to PAM50 via a nonlinear slice-wise transformation using *sct_register_to_template* (SCT).

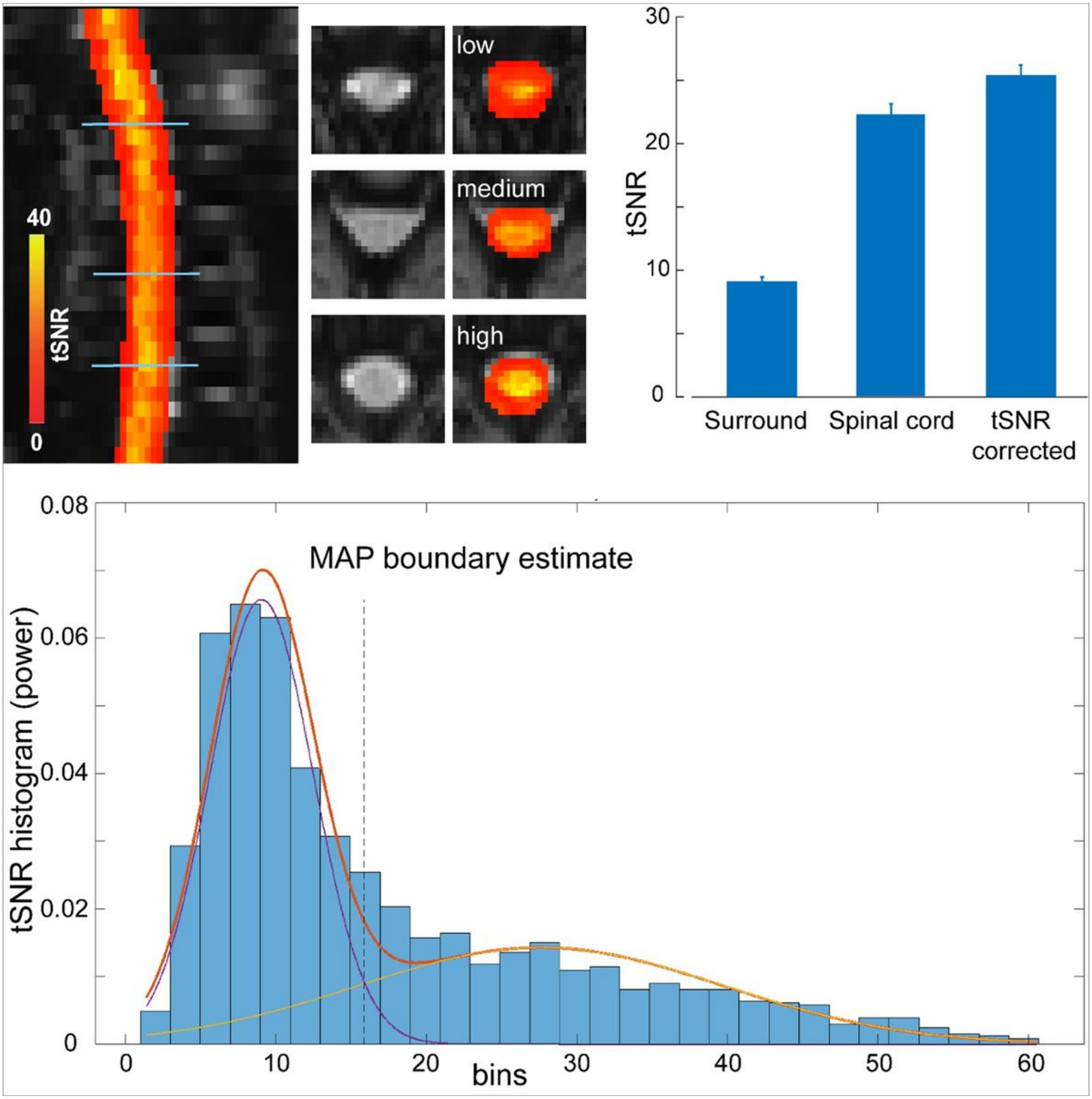

**Figure 3**

**The tSNR threshold estimation procedure.** Top left: the tSNR map in the spinal cord and the surround mask for a representative participant. Some slices around the disk level include low tSNR voxels in the spinal cord due to exacerbated susceptibility artifacts. Top right: the average tSNR values across all participants inside the spinal cord mask, the surround mask (containing mainly CSF), and the spinal cord tSNR-corrected mask. Bottom: the distribution of the tSNR values inside the spinal cord and surround mask for a representative participant, fitted using a Gaussian mixture distribution model using two components. The dashed line represents the *tSNR threshold,* defined as the Maximum a Posteriori (MAP) estimate of the optimal boundary between the two Gaussian distributions. tSNR: temporal Signal-Noise Ratio

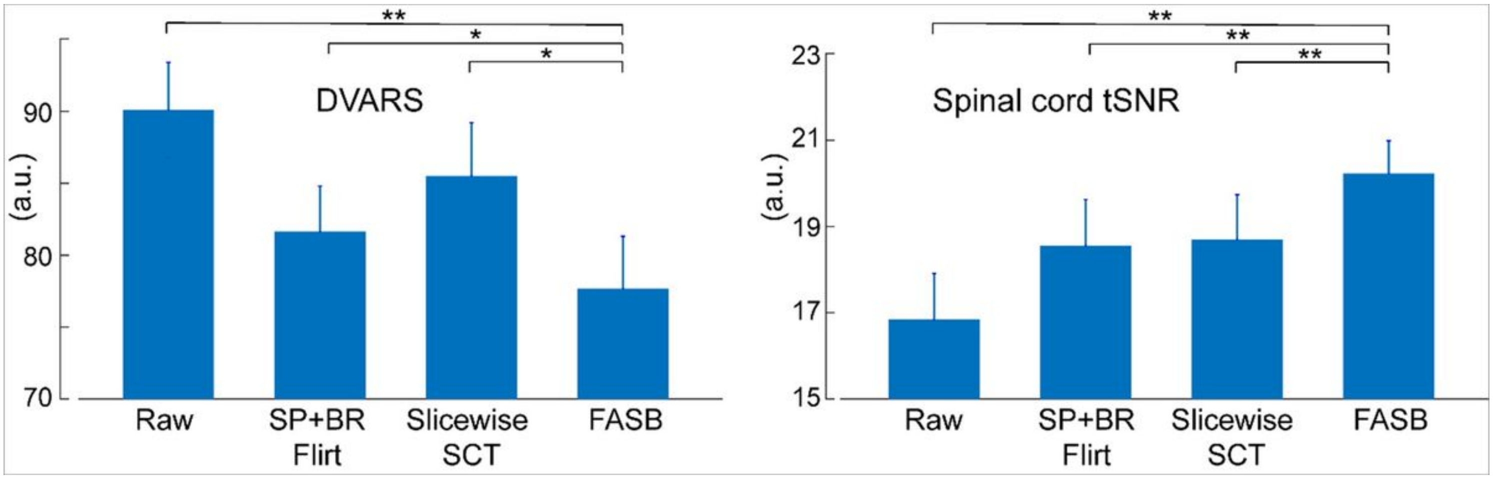

### Figure 4

**Performance of various motion correction procedures for spinal cord EPI data.**Left and right panels, respectively, show the average performance across participants of different motion correction procedures as evaluated by DVARS and tSNR in the spinal cord mask. The combined linear and non-linear procedure (FASB) outperforms the other methods [linear (SP+BR Flirt), and nonlinear (Slice-wise SCT)] in terms of both DVARS and tSNR. "Raw" reports results without motion correction. * and ** indicates $p < 0.05$ and $p < 0.01$, repeated-measures ANOVA. Error bar: sem. tSNR: temporal Signal-Noise Ratio, SP: Spinal cord; BR: Brain

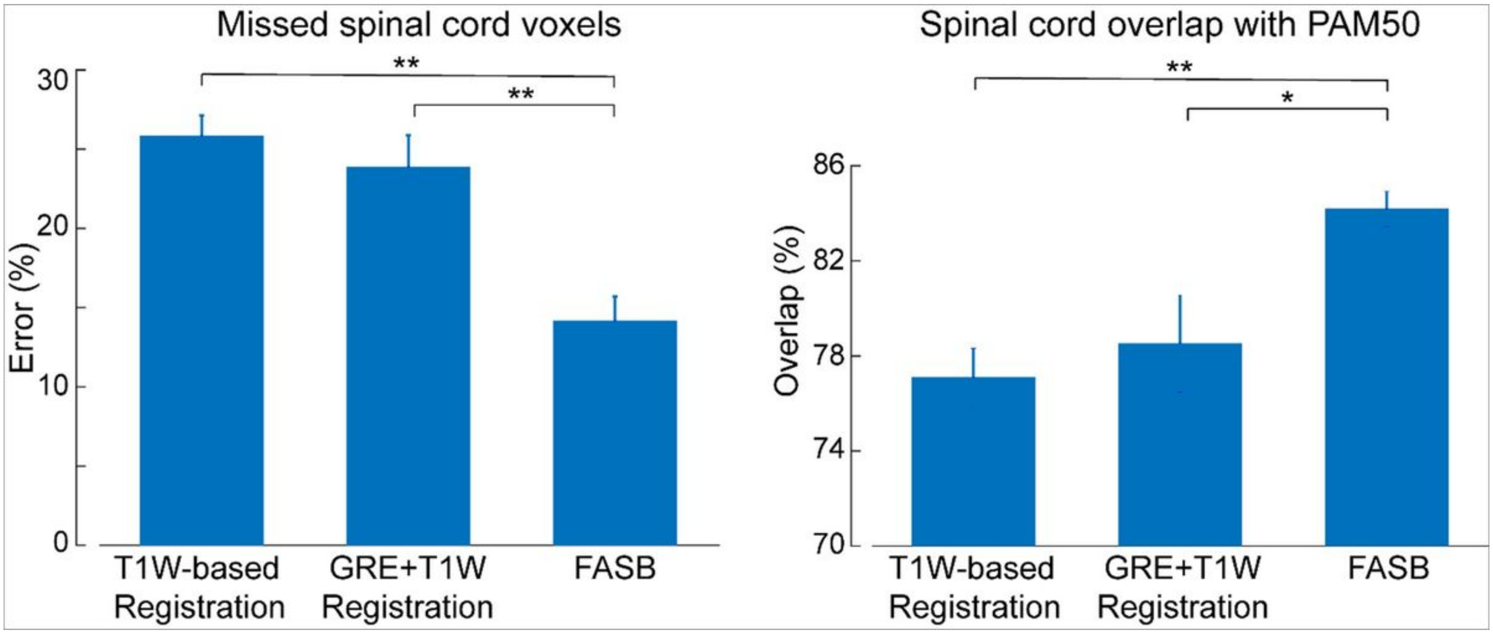

Figure 5

**Performance of various registration methods to align the EPI spinal cord image to the template.** Left and right panels, respectively, show the average performance across participants of different registration methods as evaluated by Error and Overlap ratios associated with the registered spinal cord mask in the PAM50 space. FASB registration method outperforms the other methods [T1w-based registration, and GRE correction+T1w-based registration] in terms of both Error and Overlap measures. * and ** indicates $p < 0.05$ and $p < 0.001$, repeated-measures ANOVA. Error bar: sem.

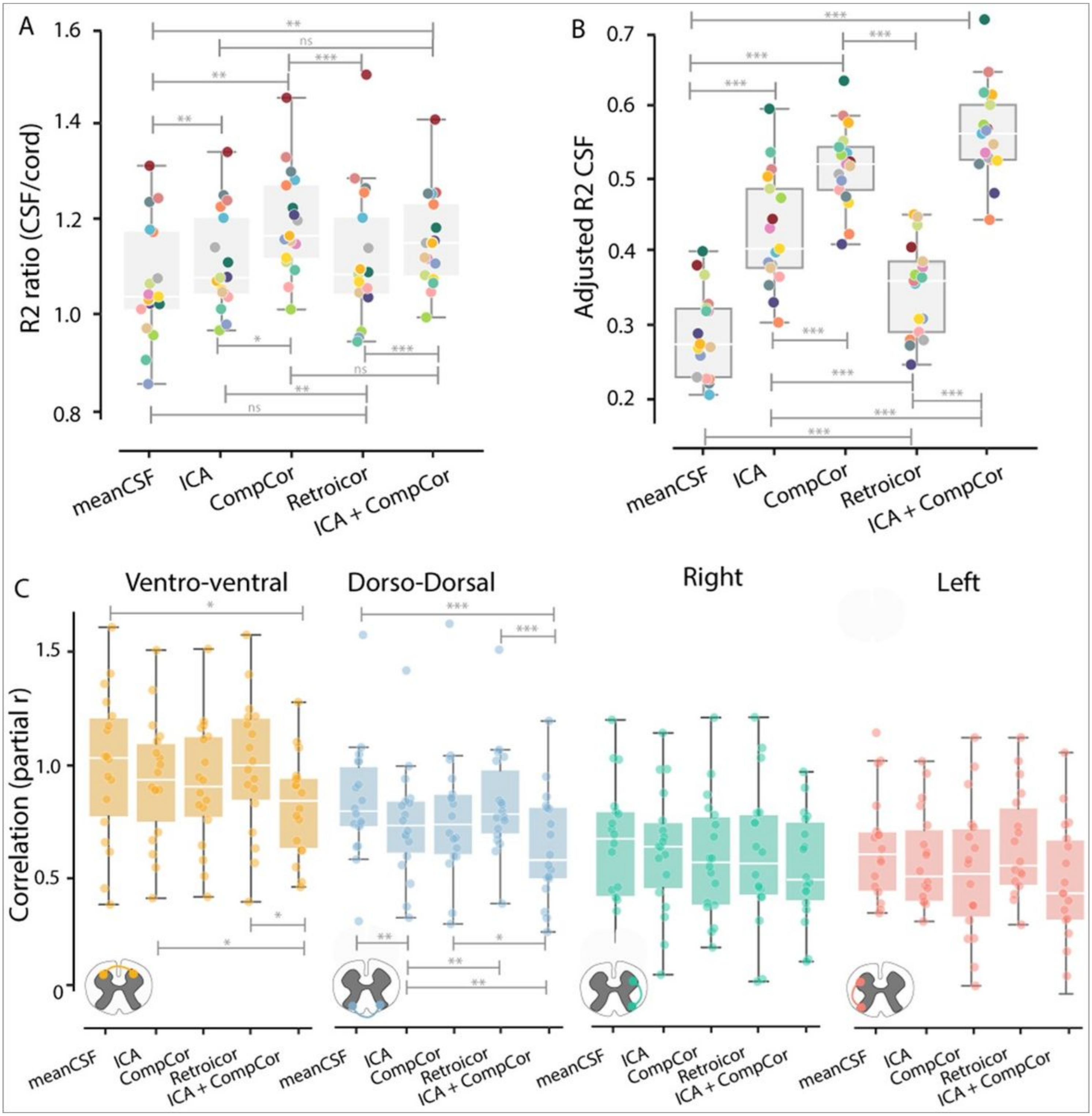

### Figure 6

**Performance of various noise removal models on the collected resting-state fMRI data. (A)** shows the ratio between the $R^2$ of CSF and spinal cord using different noise removal models, averaged across participants. *CompCor* outperforms the other models in terms of $R^2$ ratio. **(B)** shows the adjusted $R^2$ ($AdjR^2$) inside the CSF mask, averaged across participants. *ICA*, *CompCor* and *ICA + CompCor* models outperform the *meanCSF* and *Retroicor* models in terms of $AdjR^2$. **(C)** shows functional connectivity between various spinal cord quadrants using various denoising models. As shown, the choice of model does not significantly impact the calculated functional connectivity results. ** indicates $p < 0.001$. Error bar: sem.

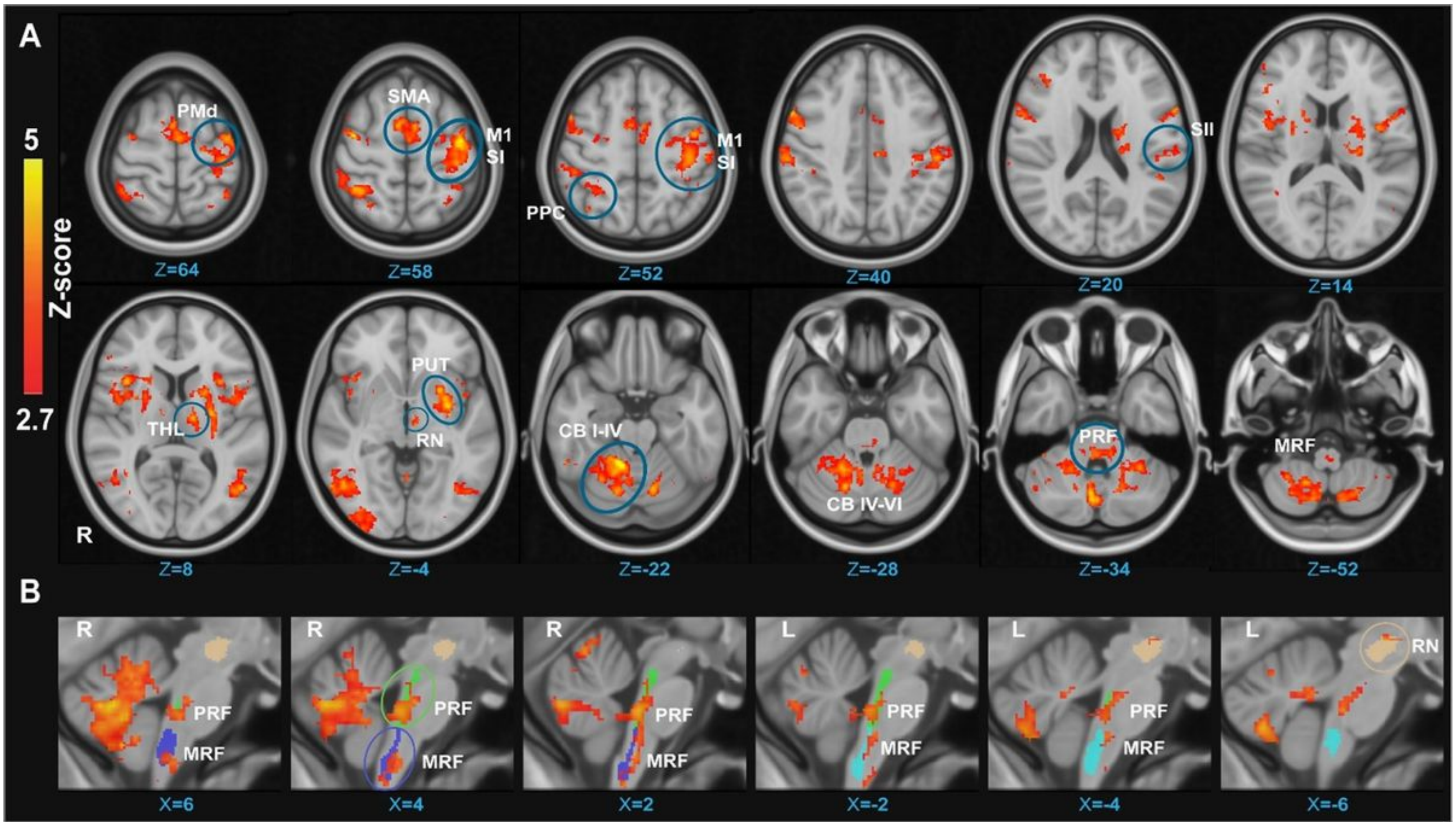

### Figure 7

**Brain activation map (in radiological view) associated with unilateral handgrip force control. (A)** Unilateral handgrip force control (right hand) results in significant activation clusters mainly in the contralateral sensorimotor cortical, thalamic, and striatal areas, as well as ipsilateral cerebellar and brainstem regions. **(B)** The zoomed-in views of sagittal slices covering the brainstem highlight significant activation clusters in the ipsilateral MRF and PRF regions. The anatomical locations of MRF and PRF are respectively highlighted in blue and green colors. PMd: dorsal premotor cortex, SMA: supplementary motor area, M1: primary motor cortex, S1: primary somatosensory cortex, SII: secondary somatosensory cortex, PUT: putamen, THL: thalamus, RN: red nucleus, CB IV-VI: cerebellum lobule IV-VI, PRF: pontine reticular formation, MRF: medullary reticular formation. R: right (ipsilateral), L: left (contralateral). All activation maps are corrected for multiple comparisons using GRF, $Z > 2.7$, $p < 0.05$.

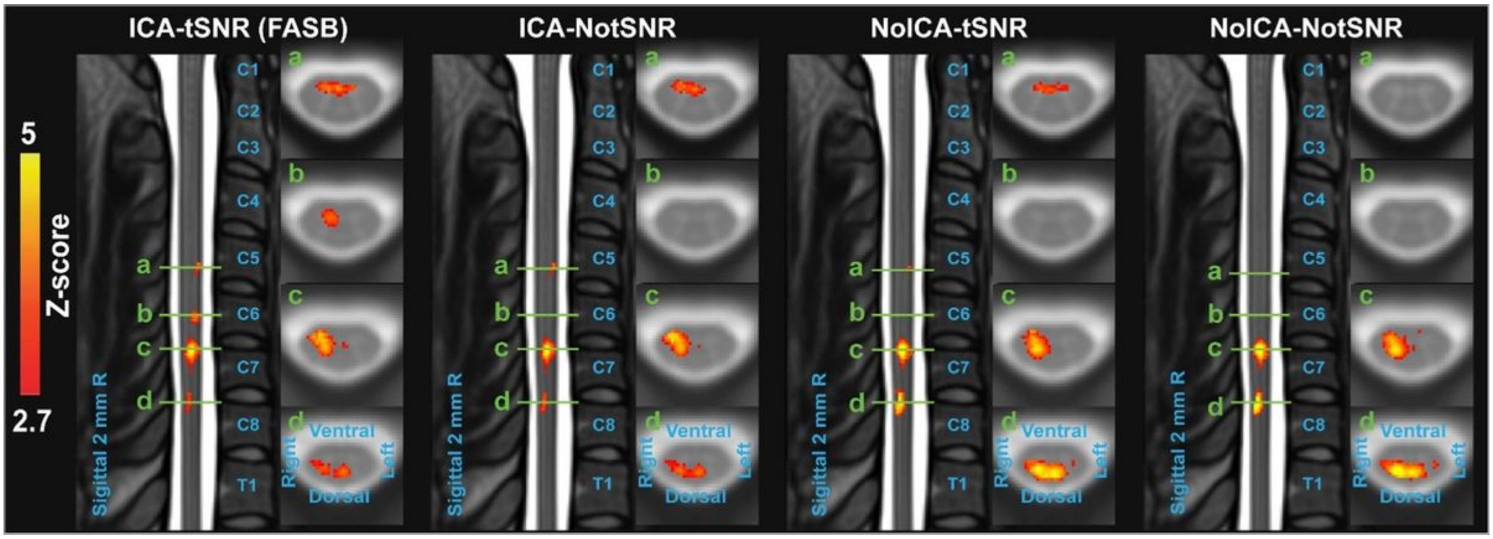

Figure 8

**Spinal cord activation map associated with unilateral handgrip force control using various analysis methods.** From left to right, each panel shows the significant activation clusters associated with unilateral handgrip force control using: FASB (ICA inclusion and tSNR correction), only ICA inclusion (ICA-NotSNR), only tSNR correction (NoICA-tSNR), and no ICA inclusion nor tSNR correction (NoICA-NotSNR). As expected, the activation clusters are located in the ipsilateral ventral horn at the lower cervical levels. The FASB method results in the greatest number of activation clusters covering the C5-C8 cervical segmental levels. All activation maps are corrected for multiple comparisons using GRF, $Z > 2.7$, $p < 0.05$. tSNR: temporal Signal-Noise Ratio, ICA: Independent Component Analysis.

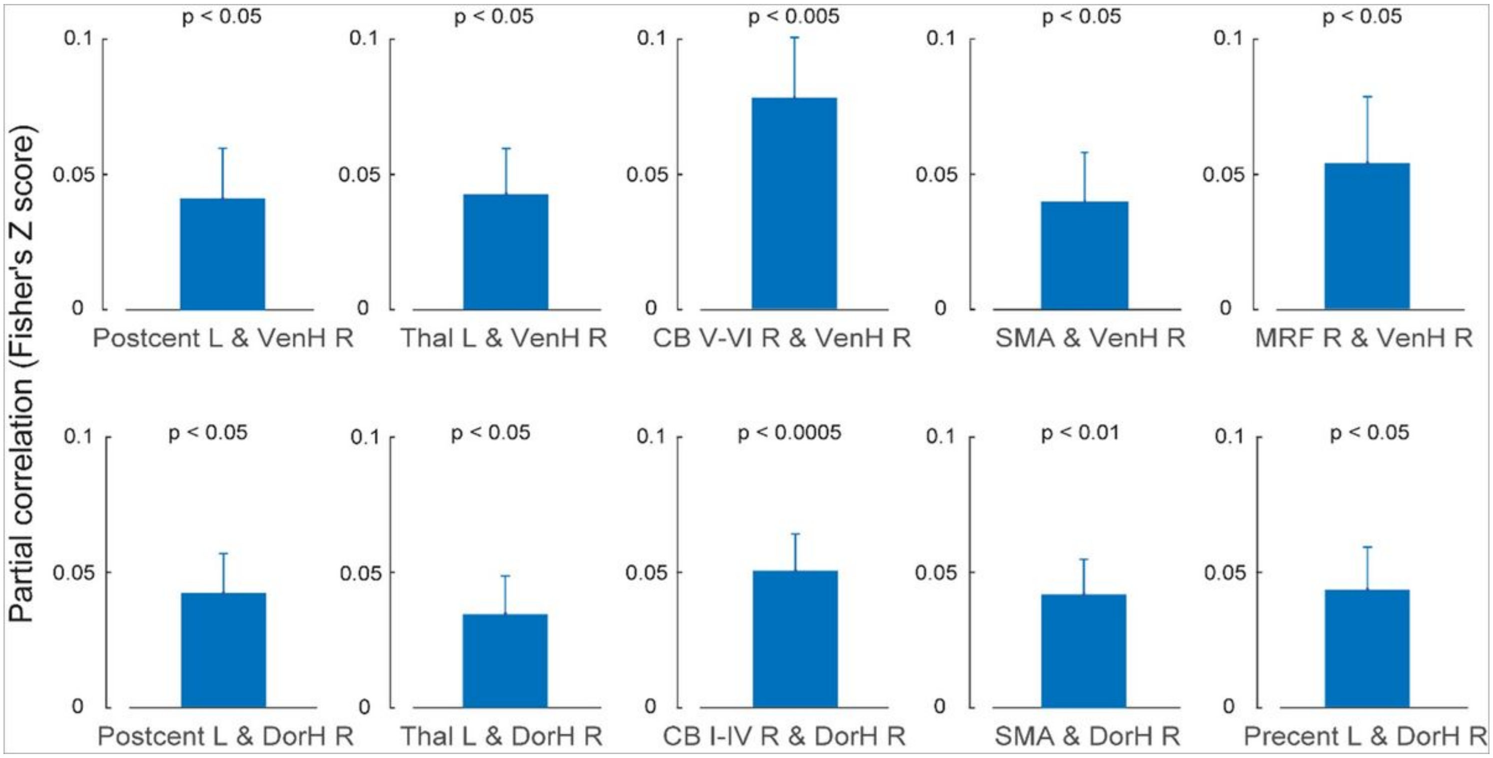

Figure 9

**Functional connectivity between various brain and spinal cord regions calculated using FASB pipeline.** Figure shows significant functional connectivity of various brain regions with the ipsilateral ventral and dorsal cervical cord (C5-C8 levels) as evaluated by partial correlation analysis during unilateral handgrip force control task. Postcent: postcentral gyrus, Precent: precentral gyrus, Thal: thalamus, CB V-VI: cerebellum lobule V-VI, SMA: supplementary motor area, MRF: medullary reticular formation, VenH: ventral horn, DorH: dorsal horn, R: right (ipsilateral), L: left (contralateral). *p*-values reports repeated measures *t*-statistics.
